# Supplementary material for: Circadian rhythm shows potential for mRNA efficiency and self-organized division of labor in multinucleate cells
Source: PLoS Comput Biol. 2021 Aug 2;17(8):e1008828. doi: 10.1371/journal.pcbi.1008828 (PMC8360590; doi:10.1371/journal.pcbi.1008828)
Supplement: S1 Appendix — (PDF) [file pcbi.1008828.s001.pdf]

S1 Appendix. Fast protein diffusion justifies treating compartmental protein concentrations as uniform

In Section 1 we stated that, since diffusion of cytoplasmic proteins is "fast" relative to the rates of protein translation and import, we can reasonably ignore diffusive mechanics, and simply assume that cytoplasmic protein concentration is at all times uniform across the syncytium. This assumption allows us to greatly reduce the number of time steps in numerical simulations. Below is a justification for this assumption.

We begin by rewriting our syncytial circadian rhythm model, but with diffusion between nuclear compartments included. Suppose that cytoplasmic proteins move into adjacent compartments at rate ξ, with reflecting boundary conditions at each end of the cell. We use the same parameter values for import, export, and decay as in [1], and set them equal to each other: $\gamma_m = \delta_m = \gamma_p = \delta_p = \nu$. The model equations read as follows:

$$\frac{dM_n^{(i)}}{dt} = \frac{\alpha}{V_n}\left(\frac{K}{K + P_n^{(i)}}\right)^r - \nu M_n^{(i)}, \quad i = 1, ..., N; \tag{S1}$$

$$\frac{dM_c^{(i)}}{dt} = \nu\left(\frac{V_n}{V_c}\right) M_n^{(i)} - \nu M_c^{(i)}, \quad i = 1, ..., N; \tag{S2}$$

$$\frac{d\mathcal{P}_c^{(i)}}{dt} = \beta M_c^{(i)} - \nu P_c^{(i)} + \xi\left(P_c^{(i-1)} - 2P_c^{(i)} + P_c^{(i+1)}\right), \quad i = 2, ..., N-1, \tag{S3}$$

$$\frac{dP_c^{(1)}}{dt} = \beta M_c^{(1)} - \nu P_c^{(1)} + \xi(P_c^{(2)} - P_c^{(1)}),$$

$$\frac{dP_c^{(N)}}{dt} = \beta M_c^{(N)} - \nu P_c^{(N)} + \xi(P_c^{(N-1)} - P_c^{(N)});$$

$$\frac{dP_n^{(i)}}{dt} = \nu\left(\frac{V_c}{V_n}\right) P_c^{(i)} - \nu P_n^{(i)}, \quad i = 1, ..., N. \tag{S4}$$

Let $m_n = V_n M_n$, $m_c = V_c M_c$, $p_c = V_c P_c$, and $p_n = V_n P_n$. Also, let $\tau = \nu t$, $\tilde{\alpha} = \alpha/\nu$, and $\kappa = V_n K$. Then we have

$$\frac{dm_n^{(i)}}{d\tau} = \tilde{\alpha}\left(\frac{\kappa}{\kappa + p_n^{(i)}}\right)^r - m_n^{(i)}, \quad i = 1, ..., N; \tag{S5}$$

$$\frac{dm_c^{(i)}}{d\tau} = m_n^{(i)} - m_c^{(i)}, \quad i = 1, ..., N; \tag{S6}$$

$$\frac{dp_c^{(i)}}{d\tau} = \frac{\beta}{\nu} m_c^{(i)} - p_c^{(i)} + \frac{\xi}{\nu}\left(p_c^{(i-1)} - 2p_c^{(i)} + p_c^{(i+1)}\right), \quad i = 2, ..., N-1, \tag{S7}$$

$$\frac{dp_c^{(1)}}{d\tau} = \frac{\beta}{\nu} m_c^{(1)} - p_c^{(1)} + \frac{\xi}{\nu}(p_c^{(2)} - p_c^{(1)}),$$

$$\frac{dp_c^{(N)}}{d\tau} = \frac{\beta}{\nu} m_c^{(N)} - p_c^{(N)} + \frac{\xi}{\nu}(p_c^{(N-1)} - p_c^{(N)});$$

$$\frac{dp_n^{(i)}}{d\tau} = p_c^{(i)} - p_n^{(i)}, \quad i = 1, ..., N. \tag{S8}$$

Now, let's estimate the rate parameter ξ. Let D be the diffusion coefficient for cytoplasmic protein and l be the length of each nuclear compartment. To reach an adjacent compartment, each protein must travel a distance of $l/2$, on average. From [2], the mean time $\overline{T}$ for a protein to be displaced by $l/2$ is

$$\overline{T} = \frac{1}{2D}\left(\frac{l}{2}\right)^2 = \frac{l^2}{8D}. \tag{S9}$$

It follows that the average rate of exchange of proteins between compartments is

$$\xi = \frac{1}{\overline{T}} = \frac{8D}{l^2}. \tag{S10}$$

The diffusivity of a protein depends on its size and the viscosity of the cytosol through which the protein diffuses; for example, (small) GFP proteins have a diffusion coefficient of approximately 33 $\mu\text{m}^2/s$ in the cells of the filamentous fungus *Aspergillus niger* [3]. However, the molecular mass of FRQ protein (108 kDa) is 4 times greater than that of GFP (27 kDA), so, assuming a globular structure, we would expect its diffusivity to be reduced by a factor of $4^{1/3}$ [2], which gives $D \approx 21$ $\mu\text{m}^2/s$. Now, assuming that $l = 5$ μm, we obtain

$$\xi \approx 6.7\ s^{-1} \approx 24000\ h^{-1}. \tag{S11}$$

Since we use $\nu = 2\pi/22 \approx 0.3\ h^{-1}$, $\epsilon := \nu/\xi$ is a very small dimensionless parameter. Multiplying both sides of Eq (S7) by ϵ, we obtain the following dimensionless system:

$$\frac{dm_n^{(i)}}{d\tau} = \tilde{\alpha}\left(\frac{\kappa}{\kappa + p_n^{(i)}}\right)^r - m_n^{(i)}, \quad i = 1, ..., N; \tag{S12}$$

$$\frac{dm_c^{(i)}}{d\tau} = m_n^{(i)} - m_c^{(i)}, \quad i = 1, ..., N; \tag{S13}$$

$$\epsilon\frac{dp_c^{(i)}}{d\tau} = \epsilon\left(\tilde{\beta}m_c^{(i)} - p_c^{(i)}\right) + \left(p_c^{(i-1)} - 2p_c^{(i)} + p_c^{(i+1)}\right), \quad i = 2, ..., N-1, \tag{S14}$$

$$\epsilon\frac{dp_c^{(1)}}{d\tau} = \epsilon\left(\tilde{\beta}m_c^{(1)} - p_c^{(1)}\right) + (p_c^{(2)} - p_c^{(1)}),$$

$$\epsilon\frac{dp_c^{(N)}}{d\tau} = \epsilon\left(\tilde{\beta}m_c^{(N)} - p_c^{(N)}\right) + (p_c^{(N-1)} - p_c^{(N)});$$

$$\frac{dp_n^{(i)}}{d\tau} = p_c^{(i)} - p_n^{(i)}, \quad i = 1, ..., N, \tag{S15}$$

where $\tilde{\beta} = \beta/\nu$. Now, setting ϵ to 0 gives the "fast" dynamics for cytoplasmic protein:

$$p_c^{(i-1)} - 2p_c^{(i)} + p_c^{(i+1)} = 0 \quad (i = 2, ..., N-1); \quad p_c^{(1)} = p_c^{(2)}, \quad p_c^{(N)} = p_c^{(N-1)}. \tag{S16}$$

Solving this system and returning to the original variables gives

$$P_c^{(1)} = P_c^{(2)} = ... = P_c^{(N)}, \tag{S17}$$

i.e., a uniform concentration of proteins across the cytoplasm of the cell. The "slow" dynamics for cytoplasmic protein are obtained by collecting $\mathcal{O}(\epsilon)$ terms in Eq (S14):

$$\frac{dp_c^{(i)}}{d\tau} = \tilde{\beta}m_c^{(i)} - p_c^{(i)}. \tag{S18}$$

In terms of the original parameters and variables,

$$\frac{dP_c^{(i)}}{dt} = \underbrace{\beta M_c^{(i)}}_{\text{translation}} - \underbrace{\gamma_p P_c^{(i)}}_{\text{import}}. \tag{S19}$$

This analysis supports our assumption that protein concentrations are kept continuously uniform between compartments.

References

1. Wang G, Peskin CS. Entrainment of a cellular circadian oscillator by light in the presence of molecular noise. Phys Rev E. 2018;97(6):062416.
2. Nelson P. Biological Physics. WH Freeman New York; 2004.
3. Bleichrodt RJ, Hulsman M, Wösten HA, Reinders MJ. Switching from a unicellular to multicellular organization in an *Aspergillus niger* hypha. mBio. 2015;6(2).
