# Supplementary material for: Circadian rhythm shows potential for mRNA efficiency and self-organized division of labor in multinucleate cells
Source: PLoS Comput Biol. 2021 Aug 2;17(8):e1008828. doi: 10.1371/journal.pcbi.1008828 (PMC8360590; doi:10.1371/journal.pcbi.1008828)
Supplement: Attachment — Submitted filename: PCOM_Neurospora_SJ.pdf [file pcbi.1008828.s002.pdf]

The manuscript “Circadian rhythm shows potential for mRNA efficiency and self-organized division of labor in multinucleate cells” investigates the circadian rhythms of multinucleate cells by developing a simple mathematical model of circadian rhythm.

Major comment.

1. The key equation of model (Eq. (5)) can be used to describe the nuclear transport of molecules from cytoplasm when every compartments have the same size. However, when their sizes are different, the equation cannot be used. For instance, let’s consider a cell with two compartments, where one compartment is 10 times larger than the other compartment. In this case, the equation (5) predicts that the concentration of nuclear protein in the larger compartment increases 10 times faster than the smaller compartment although cytoplasmic protein concentration is the same regardless of compartments. Thus, it does not make sense that larger compartment leads to higher accumulation of proteins in the nucleus. This raises the question for the major results about the effect of different sizes of compartments. Furthermore, even we accept that Eq. 5 can be used to describe the situation with compartments with different sizes, in Table 2 and Fig. 8, the change of the size of one compartment as 1.1/1.2/1.3… also changes the total compartment sizes (i.e. sum of two compartments). Thus, in table 2 and Fig. 8, not only the compartment size ratio, but also total compartment size are changed. Please, use the same total compartment volume and change the fraction of two compartment in the same total volume. Then, the relationship between the fraction of compartment and fraction of total mRNA transcribed or the quality factor q need to be investigated.
2. As diffusion is assumed to be fast (reasonable according to the detailed estimation), the mean-field approximation of cytoplasmic proteins are used (Eq. 4). The same approach has been widely used to investigate the coupled circadian clocks in SCN of mammals (10.1016/j.bpj.2014.02.039, Ref 10-17 in this work and more). In particular, 10.1016/j.cell.2007.02.047 (Fig. 7) also shows that the presence of coupling leads to more robust circadian clock. Please discuss the relationship between the finding of this manuscript and finding of previous modeling studies for coupling in mammalian circadian clock.

3. The relationship between quality factor and the number of nuclei is investigated in Fig. 4. It would be interesting to investigate the relationship between frequency and the number of nuclei. Previously, it was shown that when protein-sequestration based model (mammalian clock) are coupled, the periods are the same regardless of coupling strength (10.1016/j.bpj.2014.02.039 and 10.1049/iet-syb.2015.0090). On the other hand, when Hill-type based model (Neurospora clock) are coupled, the periods considerably change depending on coupling strength (10.1016/j.bpj.2014.02.039 and 10.1049/iet-syb.2015.0090), which has been recently confirmed experimentally (10.1038/srep35828). Thus, it would be nice to investigate whether the number of nuclei, which affects the coupling, changes frequency or not.

Minor comment

1. Introduction L30-39: Previous math models of Nuerospora clock including Dovzhenok et al are described. "Modeling reveals a key mechanism for light-dependent phase shifts of Neurospora circadian rhythms, Biophy J (2018)" is the most recent work, which improves Dovzhenok et al.

2. It would be nice to merge Fig.1-3 as Fig. 1 (optional). This will increase the readability.

3. At line 130, the values of some parameters (e.g, $V_c = 2, r = 5$) are not provided.

4. Notation is duplicated. In the equation (3), $P_c^{(i)}$ stands for the concentration of cytoplasmic protein, however, it also stands for the number of cytoplasmic protein molecules in the equation (7). Please use different fonts for the concentration and the number of molecules.
