# Supplementary material for: Circadian rhythm shows potential for mRNA efficiency and self-organized division of labor in multinucleate cells
Source: PLoS Comput Biol. 2021 Aug 2;17(8):e1008828. doi: 10.1371/journal.pcbi.1008828 (PMC8360590; doi:10.1371/journal.pcbi.1008828)
Supplement: Attachment — Submitted filename: responseToReviewers.pdf [file pcbi.1008828.s003.pdf]

(PCOMPBIOL-D-21-00232) - [EMID:3f36791457b7fbcf]
Response to Reviewers

Dear Drs. Alber and Cannon,

Thank you very much for the opportunity to submit a revised draft of our manuscript "Circadian rhythm shows potential for mRNA efficiency and self-organized division of labor in multinucleate cells." We appreciate your and the reviewers' time and effort to provide constructive feedback and insightful comments on our manuscript. We have incorporated most of the suggestions made by the reviewers, and are grateful for their close reading of the manuscript. Changes are highlighted in red text in the marked file of our revised manuscript. Below is a point-by-point response to the reviewers' concerns. Line numbers and page numbers in the responses will refer to the revised manuscript. Thank you for your consideration.

Reviewer #1:

1. *"The key equation of the model (Eq (5)) can be used to describe the nuclear transport of molecules from cytoplasm when every compartment has the same size. However, when their sizes are different, this equation cannot be used."*

   Suppose we have two cytoplasmic compartments with volumes V_1 and V_2, where $V_1 > V_2$. Now consider the discretized version of Eq (3) for the *number* of cytoplasmic proteins p_c in each compartment:

   $$p_c^{(i)}(t + \Delta t) = p_c^{(i)}(t) + \Delta t(\beta m_c^{(i)} - \gamma_p p_c^{(i)}), \quad i = 1, 2.$$

   We see that, at each time step, a total of $(\gamma_p \Delta t)p_c^{(i)}(t)$ proteins are imported into their local nucleus. Now, suppose that each compartment has the same protein concentration X at time t. Since $p_c^{(i)}(t) = V_i X$ for $i = 1, 2$, we have

   $$(\gamma_p \Delta t)p_c^{(1)}(t) > (\gamma_p \Delta t)p_c^{(2)}(t).$$

   Hence, more proteins are imported into nucleus 1 than into nucleus 2 (V_1/V_2 times more proteins, specifically). It follows that the change in concentration from protein import in compartment 1 is (V_1/V_2 times) greater than the change in compartment 2, assuming that the nuclear volumes are equal. This scaling is accounted for by the V_c/V_n term in Eq (5).

   Our assumption is that a fixed fraction of proteins from the local cytoplasm of each nucleus are imported at each time step. This assumption probably breaks down for very large cytoplasmic volumes because proteins that are far away from their local nuclei are unlikely to be imported. Hence, when considering dramatically different compartment sizes, one should probably use a saturating import rate. However, since we never consider greater than a 2:1 compartment size ratio in our analysis, we used a constant import rate for simplicity.

2. *"In table 2 and Fig. 8, not only the compartment size ratio, but also total compartment size are changed. Please, use the same total compartment volume and change the fraction of two compartment in the same total volume."*

   Thank you for this comment. However, please note that spatial dynamics are not directly included in our model because our "fast" diffusion approximation. Since cytoplasmic proteins are uniformly distributed throughout our model syncytium, it is only the relative size of compartments, not the total length of the cell, that affects model behavior. That being said, we agree that it makes sense to compare the percentage of cell volume occupied by each compartment to the percentage of mRNAs transcribed in each compartment. We therefore added an additional column to Table 3 (Table 2 in

original manuscript) that gives the fraction of the total cytoplasmic volume occupied by compartment 2 (see top of page 10).

3. *"Please discuss the relationship between the finding of this manuscript and finding of previous modeling studies for coupling in mammalian circadian clock."*

   Thanks very much for this point — we agree that a review of these previous studies in connection to our work is a necessary addition to our manuscript. We have added a paragraph in the Discussion on this topic (lines 323-332).

4. *"It would be interesting to investigate the relationship between frequency and the number of nuclei...It would be nice to investigate whether the number of nuclei, which affects the coupling, changes frequency or not."*

   Thank you for this suggestion, and for directing our attention to previous studies on this topic. We found (in agreement with Kim (2014) and Kim et al. (2016), as well as experimental results from Deng et al. (2016)), that the dominant frequency increases as coupling strength (i.e., number of nuclei in our context) increases. We've added discussion on this topic to Section 2.3.1 (lines 185-190) and also added an additional table (Table 2, top of page 8) to convey these results.

5. *"Previous math models of Neurospora clock including Dovzhenok et al are described. 'Modeling reveals a key mechanism for light-dependent phase shifts...' is the most recent work, which improves Dovzhenok et al."*

   We agree that it is very appropriate and relevant to mention the work of Bellman et al. (2018). We have added a sentence describing this work to the paragraph from the Introduction that discusses previous modeling (lines 42-45).

6. *"It would be nice to merge Fig. 1-3 as Fig. 1 (optional)."*

   We carefully considered this optional suggestion, but decided against it because Figs 1-3 (Figs 2-4 in the revision) each convey different information. Fig 1 is comparing deterministic to stochastic results, Fig 2 displays time courses for mRNA, while Fig 3 displays the power spectrum for nuclear *protein*. We felt that a combined figure would need an impractically long legend to explain what each panel of the figure represented.

7. *"At line 130, the values of some parameters...are not provided."*

   We thank the reviewer for noticing this omission. We have added $V_c = 2$ pL, $r = 5$ to the list of parameters (lines 139-140).

8. *"Notation is duplicated...Please use different fonts for the concentration and number of molecules."*

   Thank you for noting this ambiguity — we have changed the notation for mRNA and protein counts to lowercase (m and p, respectively) and added a clarification (lines 130-131). This change has been reflected on all of the relevant figures. Note that this notation is now consistent with the notation in the Appendix.

Reviewer #2:

1. *"I would have appreciated to see a scheme, i.e. a picture representation of the compartments together with the molecular processes inside those and between them, which define the model."*

   Thank you for this excellent suggestion. We have added an additional figure to the manuscript (Fig 1), which is a model schematic of all the dynamics in a compartment, and also depicts transfer of cytoplasmic proteins between compartments.

2. *"While proteins are allowed to diffuse between different compartments, Eq 3 does not seem to include the situation that a protein from compartment i, but now due to rapid diffusion being in compartment j can diffuse into the nucleus of compartment j...Eq 3 needs to be changed such that import of proteins from neighboring compartments can be included."*

   We thank the reviewer for raising this concern. Please see Eq (4), which describes the redistribution of proteins at every time step. Hence, the situation you describe is included in the dynamics for cytoplasmic protein, even if it doesn't directly appear in Eq (3).

3. *"mRNA's are not allowed to diffuse into other compartments. Why is that? From a molecular mass perspective clock-mRNA (frq-mRNA) may diffuse at least as fast as proteins."*

   In "Clustered nuclei maintain autonomy and nucleocytoplasmic ratio control in a syncytium" by Dundon et al. (2016), the authors show that cyclin mRNAs tend to localize around nuclei. Although mRNAs are typically larger than the proteins they encode for, the most severe limitations upon diffusion arise from the formation of mRNA-protein droplets within the cell. We cite Dundon et al. (2016) as evidence for omitting mRNA diffusion from the model (see the paragraph directly before Eq (1), on page 4, and added text clarifying the mechanism of mRNA confinement). However, although mRNA distributions are useful as readouts of nuclear activity, free "fast" diffusion of proteins means that our results would not be affected by allowing mRNAs to diffuse. mRNAs only decay in cytoplasm in our model — they are not imported back into the nucleus. Together with our fast diffusion assumption for proteins, this means that mRNA diffusion does not actually affect model behavior.

4. *"Please note that the mRNA is exported from the nucleus into the cytosol where protein synthesis of proteins (for example FRQ) occurs. It should also be noted that the FRQ protein in Neurospora crassa is present as a dimer. This dimer formation may have some influence when considering heterokaryons."*

   We thank the reviewer for highlighting the need to separate the roles of transcription and export in the model of cytoplasmic mRNA abundances. Both terms are present in our model (see Eq (1) and (2)). It has recently been suggested that slow mRNA export may play a role in filtering fluctuations in mRNA (Singh and Bokes, 2012), and it might be that this is the effect that the reviewer is alluding to. However, in order to not create too much distance between our syncytial model and the uninucleate model from which it is derived, we have retained the default mRNA export rate within our model, and added text to the discussion about the importance of measuring the export rates to make quantitative predictions about real circadian clocks (lines 349-351).

   The reviewer also correctly notes that our model ignores post-translational protein modification — the production of dimers or complexes with other molecules. We agree with the reviewer that the facility with which *Neurospora* heterokarya can be constructed by fusion allows for perturbations of networks that span across nuclei, such as circadian rhythm, and we have added commentary in our discussion on the possibilities for future work in that direction (lines 351-356). We thank the reviewer for suggesting that we add this important context.

5. *"Table 1: The change of alpha by four-orders of magnitude should have a significant effect on the period length of the oscillator when only alpha is changed. What is the effect of alpha on the period?. While one can agree on the change on q, what does such a calculation mean? Are in the model other parameters changed to keep the period within the circadian range when alpha is increased?"*

   We thank the reviewer for this astute observation. Changing α does in fact affect the period length, though not dramatically. Peak period varies from 25.2 h for $\alpha = 180000\ h^{-1}$ to 27.8 h for $\alpha = 18\ h^{-1}$. Since both of these period lengths are in the vicinity of a day, we did not deem it necessary to

alter other parameters to keep the period in the circadian range. We added a note on these findings to the end of section 2.2 (lines 167-170).

6. *"Abstract, line 4: nuclei are capable of autonomous behavior. What kind of autonomous behavior?"*

   Asynchronous nuclear division is perhaps the best understood form of nuclear autonomy. In particular, even among genetically homogeneous *A. gossypii* nuclei, nuclei divide asynchronously. Even richer dynamics have been found in heterokarya, including selection for stable nucleotype proportions and even patterns of cyclic dominance. We have expanded the introduction to add appropriate references and context (lines 7-9).

7. *"Why should Poisson noise be considered? Are there other types of noise that need to be excluded?"*

   Poisson noise arises from random waiting times for events to occur in our model, including transcription, translation, import, export, and decay. Because we are studying circadian rhythm with very low mRNA copy numbers, Poisson noise in transcription events has a dramatic influence on model behavior for each stochastic simulation. Another possible source of noise is from transcriptional bursting. Although this effect has been considered in previous models of stochastic noise in gene expression, to prevent over-engorgement of this paper and to remain close to an existing model of cellular circadian rhythm, we leave its discussion for further work.
